# Date fruit dataset for intelligent harvesting

**DOI:** 10.1016/j.dib.2019.104514

**Published:** 2019-09-18

**Authors:** Hamdi Altaheri, Mansour Alsulaiman, Ghulam Muhammad, Syed Umar Amin, Mohamed Bencherif, Mohamed Mekhtiche

**Affiliations:** a Department of Computer Engineering, College of Computer and Information Sciences, King Saud University, Riyadh 11543, Saudi Arabia; b Center of Smart Robotics Research, College of Computer and Information Sciences, King Saud University, Riyadh 11543, Saudi Arabia

**Keywords:** Date fruits, Visual dataset, Robotic harvesting, Machine vision, Fruits classification, Visual yield estimation

## Abstract

The date palm is one of the most valuable fruit trees in the world. Most methods used for date fruit inspection, harvesting, grading, and classification are manual, which makes them ineffective in terms of both time and economy. Research on automated date fruit harvesting is limited as there is no public dataset for date fruits to aid in this. In this work, we present a comprehensive dataset for date fruits that can be used by the research community for multiple tasks including automated harvesting, visual yield estimation, and classification tasks. The dataset contains images of date fruit bunches of different date varieties, captured at different pre-maturity and maturity stages. These images cover multiple sets of variations such as multi-scale images, variable illumination, and different bagging states. We also marked date bunches for selected palms and measured the weights of the bunches, captured their images on a graph paper, and recorded 360° video of the palms. This dataset can help in advancing research and automating date palm agricultural applications, including robotic harvesting, fruit detection and classification, maturity analysis, and weight/yield estimation. The dataset is freely and publicly available for the research community in the IEEE DataPort repository [1] (https://doi.org/10.21227/x46j-sk98).

**Table:** Specifications Table

Subject	Computer Science, Machine Intelligence
Specific subject area	Machine vision; Automated harvesting; Image classification; Visual yield estimation.
Type of data	Tables (.XLSX)
	Images (.JPG)
	Videos (.MTS)
	Texts (.TXT)
How data were acquired	Three color cameras (Sony HDR-CX405, Canon EOS-600D, and Canon EOS-1100D); Two weight scales (Citizen CTB 300 and Bizerba BS 200).
Data format	Raw and Analyzed
Parameters for data collection	The data were collected in a natural orchard environment. The images and videos were taken under different natural daylight conditions: in the morning (5:00–11:00) or afternoon (3:00–5:00). The data of individual dates were acquired after harvesting under laboratory conditions at 18 °C.
Description of data collection	This data is divided into two sets. The first set consists of images of date bunches that were taken using color cameras from different angles and scales during one season in six imaging sessions over the period of Jun–Sep 2016. The second set contains images, videos, and weights that were acquired during the harvesting period of Barhi dates. At the beginning of this period, date bunches were marked on trees. Then, images and videos were collected. After harvesting, the images of these bunches were captured in front of a graph paper, and their weights were recorded.
Data source location	Center of Smart Robotics Research, King Saud University, Riyadh, Saudi Arabia. The date orchard located 25 km Northwest of Riyadh.
Data accessibility	Repository name: IEEE DataPort Data identification number: 10.21227/x46j-sk98 Direct URL to data: https://doi.org/10.21227/x46j-sk98

**Table:** 

**Value of the data** • Advanced agriculture automation such as robotic harvesting can significantly increase quality and yield and reduce production costs and delay. This dataset is a contribution to agriculture automation research. • The dataset advances the machine vision research of date fruit in pre-harvesting and harvesting stages. To the best of our knowledge, this is the only publicly available dataset for date fruit pre-harvesting and harvesting applications. • The date dataset consists of images, videos, and weight measurements, which can be used for various machine vision applications for date fruit in orchard environments, including automated harvesting, fruit detection and segmentation, classification, maturity analysis, and weight/yield estimation. • The dataset has multiple sets of variations that reflect the challenges in agriculture environments and date orchards, which is essential for building a reliable and robust machine vision.

## 1 Data

**Fig. 1:**
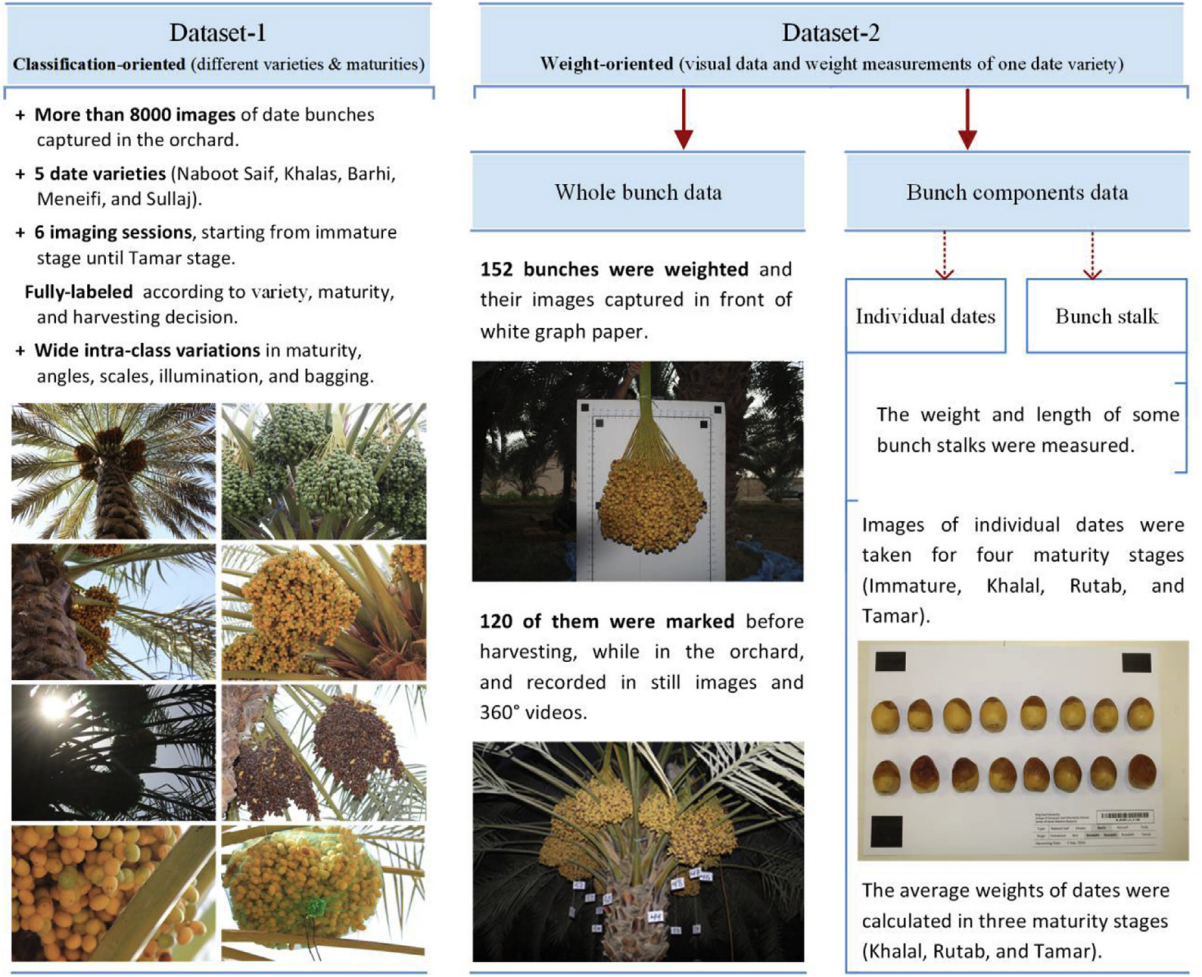
A brief description of the date fruit datasets.

The date fruit dataset was created to address the requirements of many applications in the pre-harvesting and harvesting stages. The two most important applications are automatic harvesting and visual yield estimation. Since date applications require different types of data with different characteristics, we built two separate datasets for the benefits of researchers in different date applications. Fig. 1 gives a brief overview of the two datasets.

The first dataset (dataset-1) contains images of date fruit bunches for different date varieties, showing various pre-maturity and maturity stages. These images cover a large degree of variabilities in order to address the challenges in the date orchard environment, such as multi-scale images, variable illumination, different angles, and diverse bagging states. Dataset-1 can be used in many applications such as automatic harvesting tasks, fruit detection, fruit recognition, maturity analysis, etc.

The second dataset (dataset-2) contains images, videos, and weight measurements to help in many applications such as yield estimation. In this dataset, we marked date bunches for selected palms, recorded 360° video for each palm, and measured their data (height, trunk circumference, total yield, number of bunches, and weight of bunches). We also captured images of each bunch from different angles before harvesting and on a graph paper after harvesting. Due to the enormous work needed and the huge amount of data, this dataset built for only one date variety. However, the proposed method for yield estimation (as a major application of dataset-2) based on one date variety can be generalized for other varieties because visual weight features, e.g. bunch area, perimeter, compactness, volume, etc., are almost invariable in different date varieties.

### 1.1 Dataset-1

**Fig. 2:**
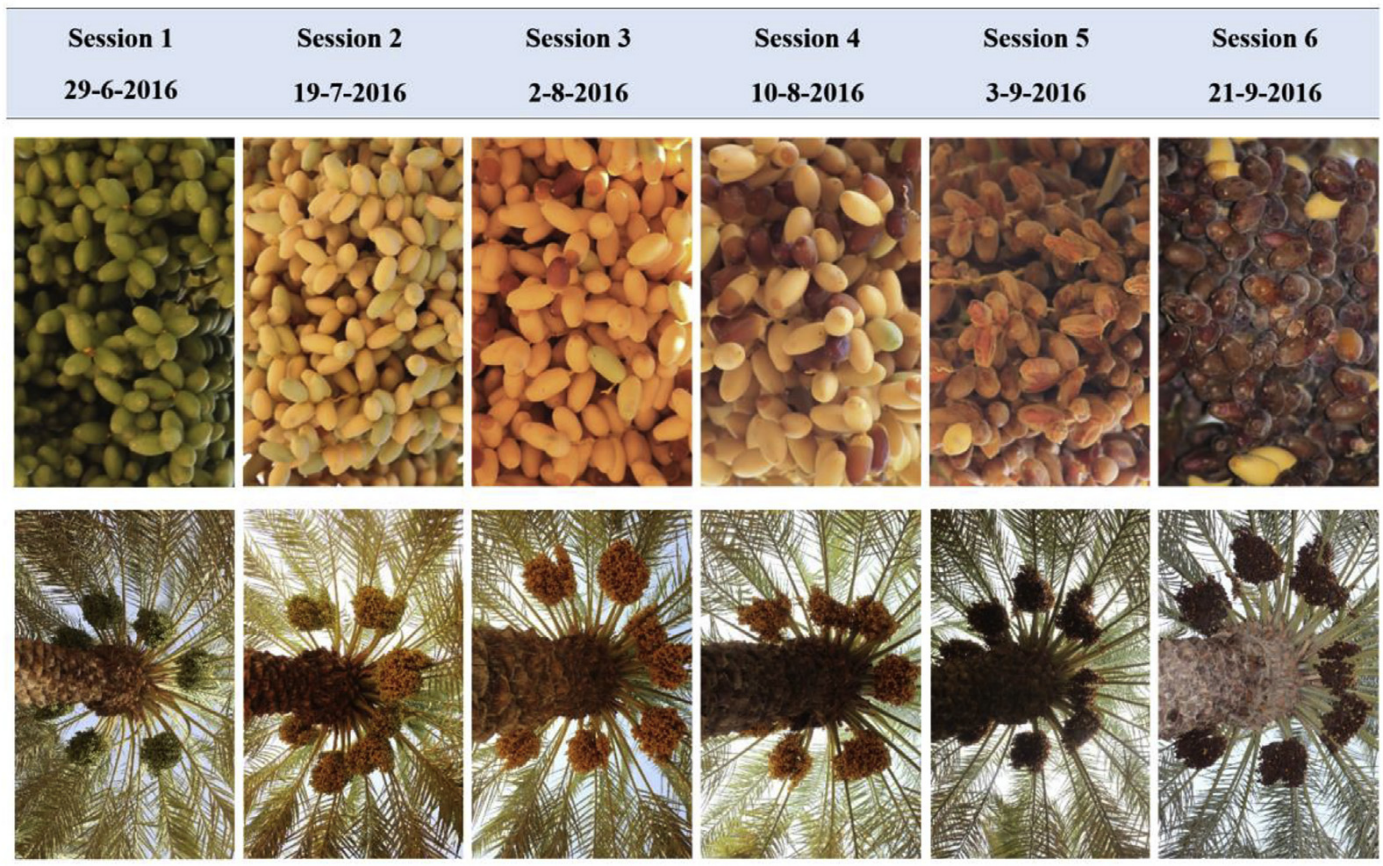
Sample images of a Sullaj date captured in six imaging sessions and covered all date maturity stages (immature, Khalal, Rutab, and Tamar).

Dataset-1 consists of 8079 images of more than 350 date bunches captured from 29 date palms. The date bunches belong to five date varieties: Naboot Saif, Khalas, Barhi, Meneifi, and Sullaj. The images of date bunches were captured using two color cameras, their models and settings are described in the next section, in six imaging sessions (recording times). The imaging sessions covered all date maturity stages: immature, Khalal, Rutab, and Tamar. Fig. 2 shows sample images of the Sullaj date fruit over the six sessions. The dates in one session may have multiple maturity stages because individual dates do not mature uniformly. The first session was when the dates are usually immature, and the last session was when the dates were in the Tamar stage. Dataset-1 was labeled according to the variety, maturity, and harvesting decision. The annotation files are available in Ref. [1]. The labeling instructions and rules are explained in detail in Ref. [2].

**Fig. 3:**
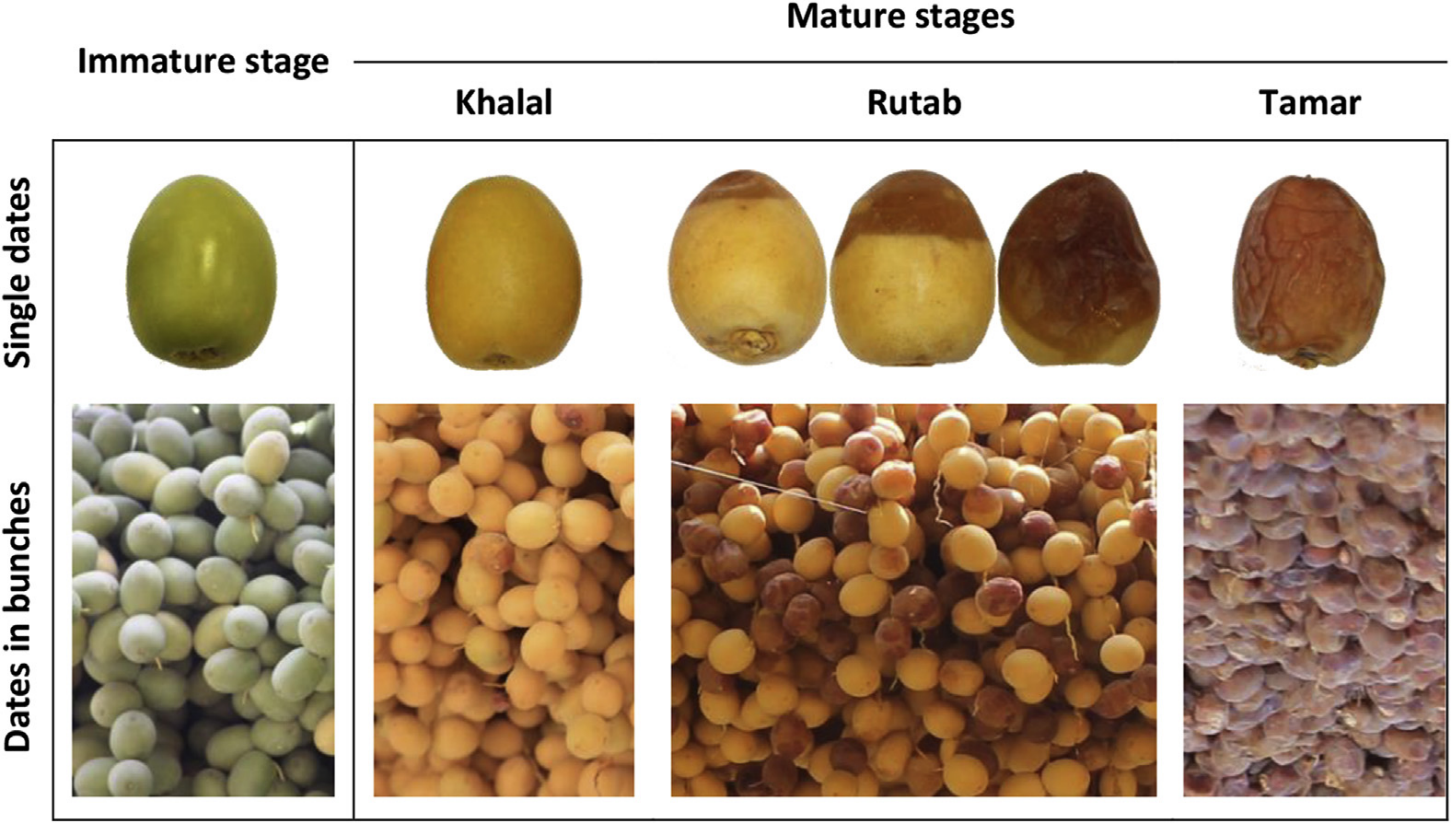
Samples of Barhi date fruit in immature and three mature stages (Khalal, Rutab, and Tamar).

Date fruits can be harvested and marketed at three stages of their development—Khalal, Rutab, and Tamar, as shown in Fig. 3. Dates in Khalal are the first in the harvesting season, where dates are considered ready for sale as fresh ripe fruit. Khalal dates have a crisp texture, hard surface, and bright yellow or red color. The Rutab stage is the second in the harvesting season. Rutab starts with the appearance of a partially browned color at the date's tip and then spreads gradually to the whole date. Rutab is delicate, highly perishable, and has short development time. Tamar is the final development stage of dates. Tamar dates have texture from soft pliable to firm to hard, and color from amber to dark brown. In the Tamar stage, dates are non-perishable, so they can be stored for a long period of time and can be consumed throughout the year. The choice for harvesting at one or another stage depends on several factors, including climatic conditions, date variety, and market demand [3].

**Fig. 4:**
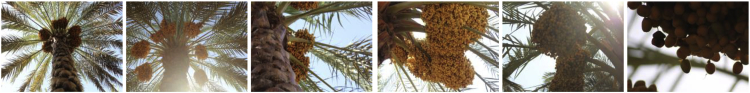
Sample images of the dataset-1 showing large variation in scales, angles, and illumination.

**Fig. 5:**
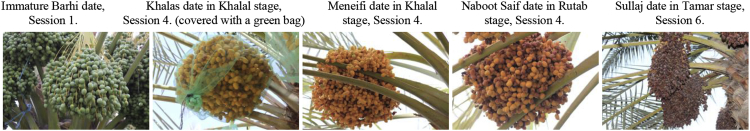
Sample images of the five date varieties in dataset-1 showing different maturity stages. Some bunches are covered with bags for their protection.

In order to build a comprehensive dataset that can address various machine vision applications and challenges for date fruits in an orchard environment, the dataset was provided with multiple sets of variations: multi-scale images, variable illumination, different date varieties, multiple maturity stages, and different bagging states, as shown in Fig. 4 and Fig. 5. Table 1 highlights these variations.

**Table 1:** The variations in dataset-1.

Variation Type	Description
Multi-scale images	Images containing part of the bunch, the whole bunch, or many bunches.
Variable illumination	The images were taken under different natural daylight conditions: in the morning (9:00–11:00) and afternoon (3:00–5:00). The images also were captured in poor illumination conditions from different camera positions relative to the sun.
Different date varieties	Five varieties: Naboot Saif, Khalas, Barhi, Meneifi, and Sullaj.
Multiple maturity stages	The images were taken in six imaging sessions, which covered all date maturity stages: immature, Khalal, Rutab, and Tamar.
Different bagging states	Bagging is performed for some high-quality date varieties for their protection. The images include some bagged date bunches.

The dataset includes multi-scale images of date bunches, as shown in Fig. 4, to increase the variation in images, which helps make the vision system robust and versatile for different harvesting methods. Some images contain part of the bunch, which helps in individual-date-based harvesting, where the vision system must detect individual dates and harvest them based on maturity. Images that contain one or many bunches can be used for bunch-based harvesting, where the vision system would detect and harvest a complete bunch based on its maturity. Some images contain whole date palms, and these can be used for applications such as palm recognition and maturity classification. The dataset also includes images for different date varieties in different maturity stages. This diversity is particularly essential for automated date fruit harvesting because date orchards usually contain different varieties of dates that are harvested and marketed at different maturity stages, e.g., Barhi dates are harvested early in the Khalal stage, whereas Khalas dates are usually left until the Tamar stage. This dataset also includes images of bagged bunches for dates of Khalas variety. These bunches were covered with green bags in the Khalal maturity stage, as shown in Fig. 5. In date orchards, bunches of some date varieties are covered (usually at the beginning of August) with a bag to protect them from dust, pests, and rain. The images of bagged bunches can be used to increase the robustness of the classification system by training the system to recognize the variety or maturity state of dates even if the bunch is bagged. It will also allow the classification of date bunches as bagged or not bagged, hence can be used for developing automatic bagging machine.

### 1.2 Dataset-2

**Fig. 6:**
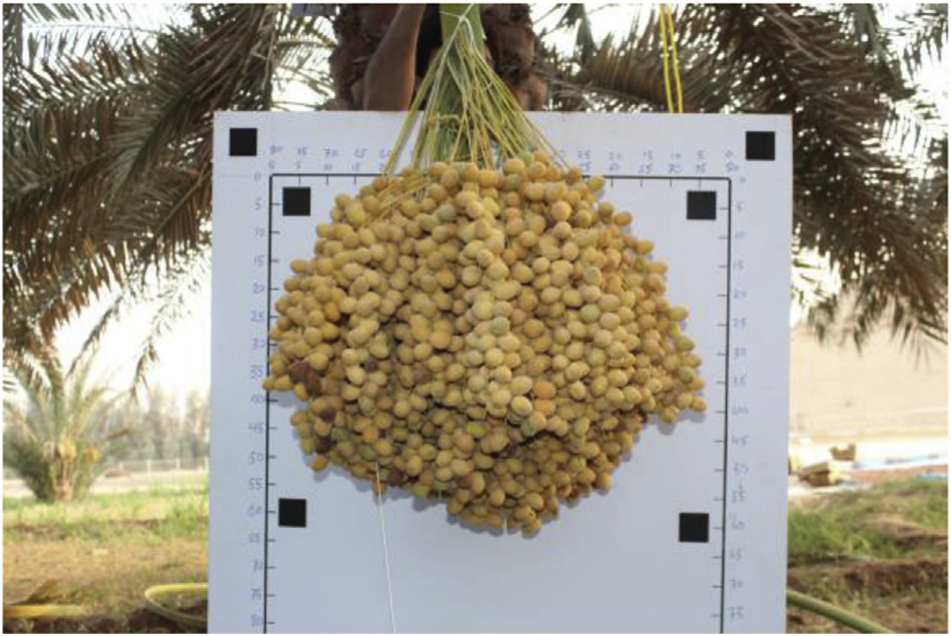
A sample of the Barhi date bunch captured in front.

**Fig. 7:**
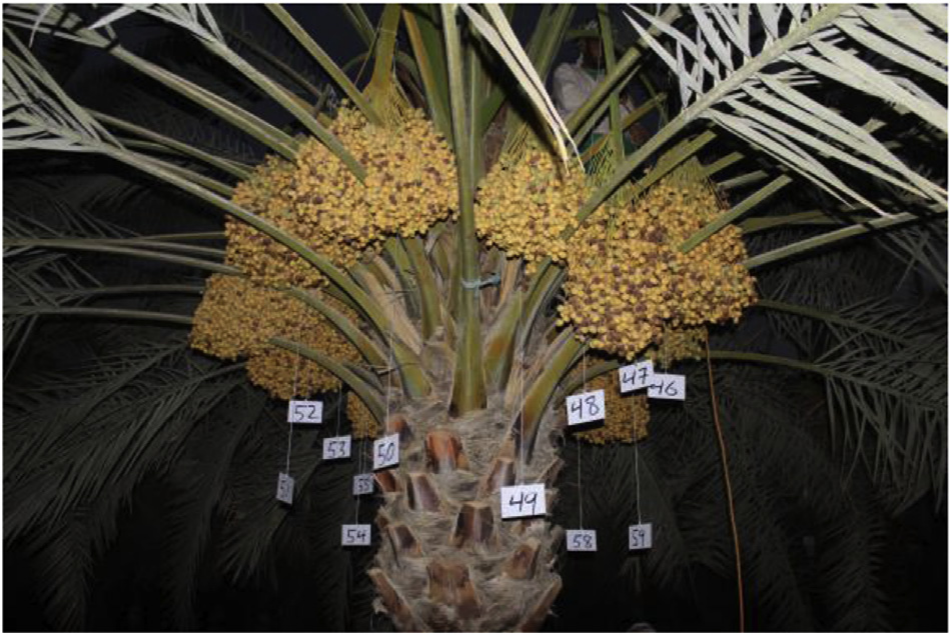
A sample of Barhi date bunches marked in a palm.

This dataset consists of 152 Barhi date bunches belonging to 13 palms. All bunches were weighted after harvesting, and their images were captured in front of a white graph paper, as shown in Fig. 6. Subset of these bunches (120 bunches from nine palms) were provided with more inclusive data: we marked the whole bunches in the palm (as in Fig. 7), captured their images from different angles before and during harvesting, recorded 360° video for each palm, and registered their characteristics (height, trunk circumference, total yield, and number of bunches).

**Fig. 8:**
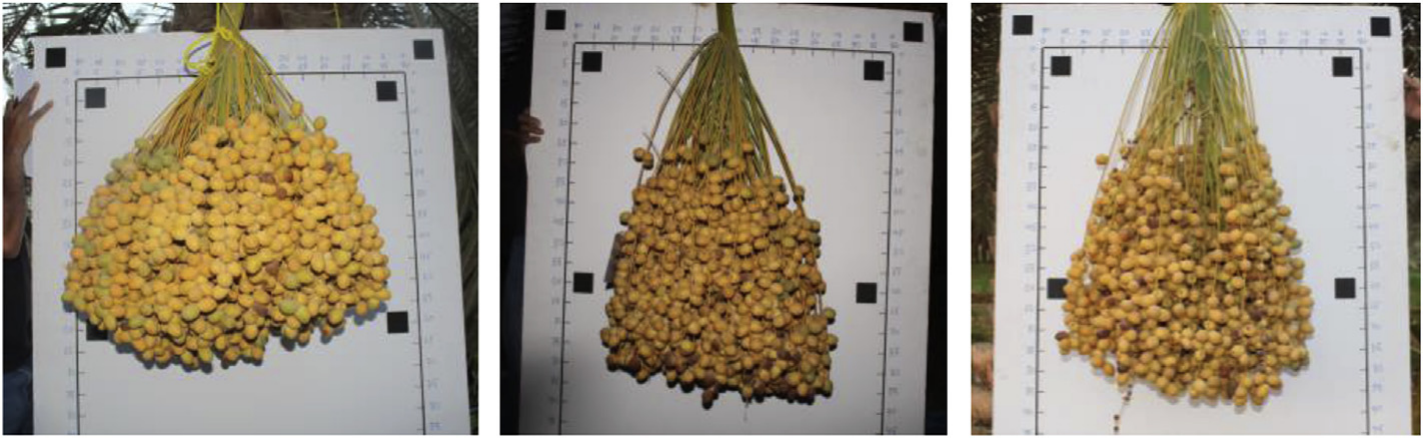
Date bunches from dataset-2 showing different levels of date compactness.

**Fig. 9:**
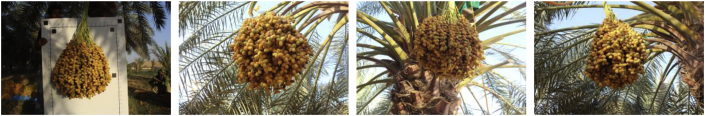
Sample images of one date bunch captured in ideal front view (with uniform background) and from other angles on the palm.

The selected 13 date palms had different heights and ages (1.7–4.85 m and 10–25 years) to maintain the variation in bunch area, perimeter, and date size as much as possible. The dataset also has variations in bunch compactness and the angle of view (Fig. 8 and Fig. 9). Including these variations are important for building an effective and reliable machine vision system for weight and yield estimation. The date compactness in a bunch is affected by the thinning process, the operation of removing some stands or dates from a bunch to enhance its quality. Therefore, different date bunches with different degrees of thinning levels were selected, as shown in Fig. 8. We also captured several images for each date bunch from various angles of view. One image was captured from a constant distance and front view-point, and other images from different variable angles, sample images are shown in Fig. 9.

The uniform background in the graph paper is used to achieve better image segmentation, where we can get a high contrast between the background and the bunch. The graph paper has six reference objects, which are uniquely identifiable by shape (square), color (black), and position (corners). The graph paper with the reference objects can be used to estimate geometric information of the bunch, i.e., area, dimensions, and perimeter.

**Fig. 10:**
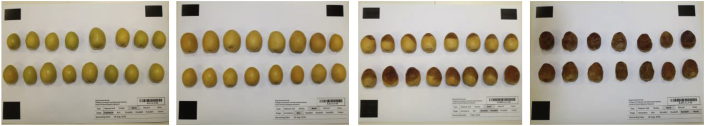
64-individual Barhi dates at the four maturity stages (16 dates per stage): immature, Khalal, Rutab, and Tamar.

In addition to the whole bunch data, other information was collected: images and weights of the bunch components (individual dates and bunch stalk). The primary purpose of this information is to estimate the bunch weight based on its components, i.e., by using dates' dimensions and weights and estimating the number of dates in the bunch. In the current dataset, images of 256 individual dates of Sullaj, Barhi, and Meneifi dates were recorded. Fig. 10 shows 64 individual Barhi dates at the four maturity stages (Immature, Khalal, Rutab, and Tamar). We also calculated the average weight of Barhi and Sullaj dates at three maturity stages (Khalal, Rutab, and Tamar). For bunch stalk, the length and weight of ten stalks were measured.

## 2 Experimental design, materials, and methods

The datasets were recorded in an orchard in Al-Ammaria, located 25 km Northwest of Riyadh, Saudi Arabia. The orchard contained more than 800 date palms of different varieties and ages.

The images of dataset-1 were taken during one season in six imaging sessions over the period of June–September 2016. The usual period between imaging sessions was 20 days, but because the Rutab stage has a short development time, we made the period during the Rutab stage 10 days (between sessions 2, 3, and 4). The images in dataset-1 were taken under different natural daylight conditions: in the morning (9:00–11:00) or afternoon (3:00–5:00). These images were captured using two RGB (Red, Green, and Blue) cameras: Canon, EOS-600D (camera-1) and EOS-1100D (camera-2), Tokyo, Japan, with variable focal length and in automatic mode. The resolutions of the cameras were 5184 × 3456 and 4272 × 2848, respectively. The orientation of the camera and the distance between the camera and the fruits depend on the height of the palm. Because the selected date palms were with different heights (1.7–6 m), these parameters were variable. For short palms, the orientation was approximately 0° and the distance approximately 3 m, but for tall palms, the orientation was from 45° to 90° and the distance was from three to 6 m.

**Fig. 11:**
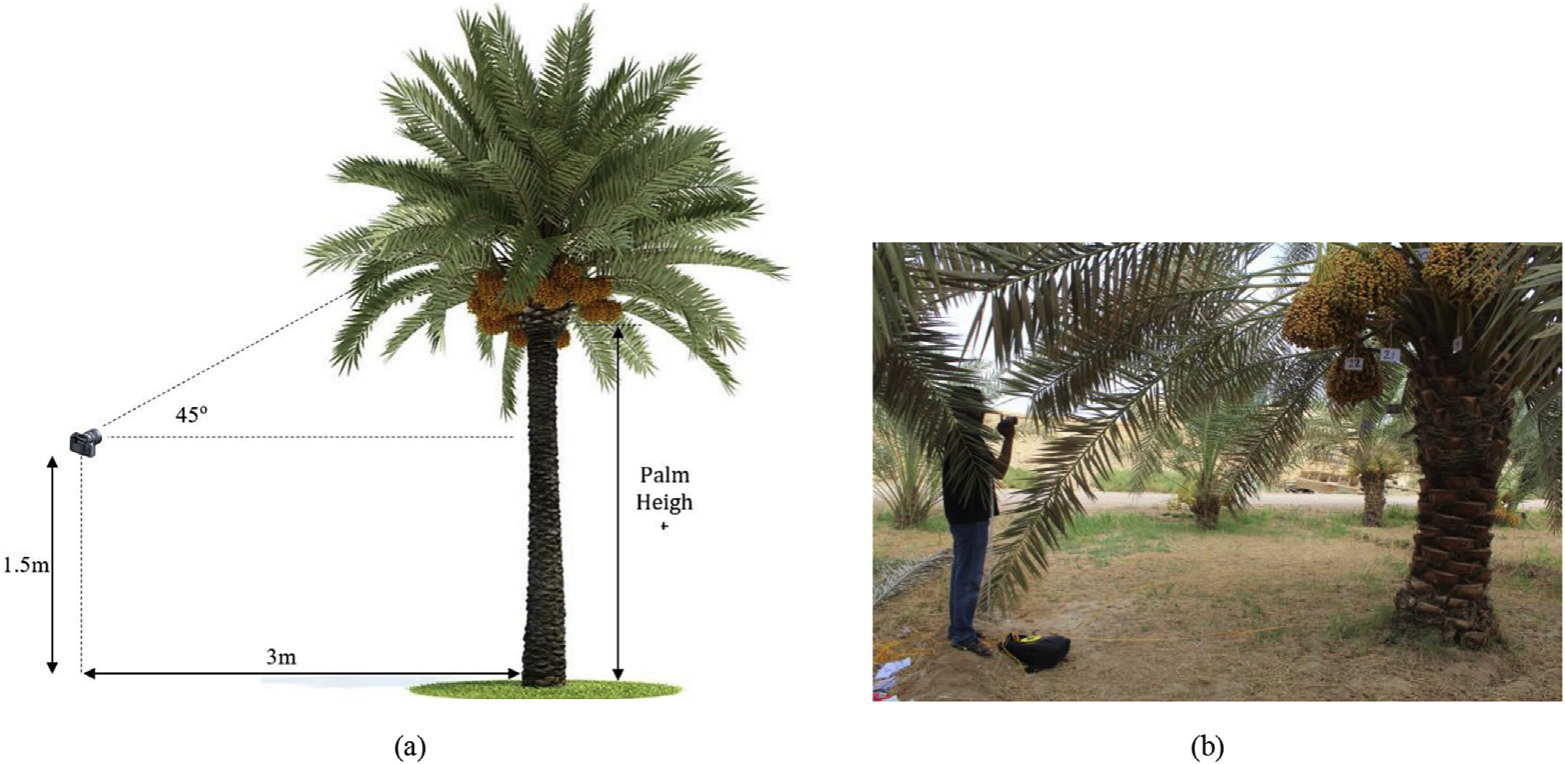
(a) Camera position setup for captured videos. The orientation of the camera depends on the palm height. Figure (a) shows a camera orientation of 45° for a three-meter high palm tree. Figure (b) shows a real example for the palm-3 (B3.K.BW); the palm height is 2.85 m, so the camera orientation was less than 45°.

In dataset-2, the images and videos were acquired in the morning (5:00–7:00 am) during the harvesting period of Barhi dates (at the Khalal stage), 16–26 August. At the beginning of this period (16 August), each date bunch was marked as shown in Fig. 7. Then, several images and videos were collected for date bunches from different angles. The images were captured using camera-1. The videos were recorded using an RGB camera (camera-3: Sony, HDR-CX405, Tokyo, Japan), in full HD resolution (1920 × 1080) and were stored in MPEG Transport Stream format (.MTS). Each video was recorded for all bunches on the palm as one continuous 360° video. The camera was moved around the palm in a circular manner at an approximate radius of 3 m and a height of 1.5 m from the ground, as shown in Fig. 11. The orientation of the camera (the angle from the horizon) ranged from zero to 80° depending on the palm heights. The characteristics of the selected palms were also registered, including palm height and trunk circumference. Palm height is the distance between the ground and the fruit, as shown in Fig. 11. The circumference of the palm trunk was measured at a height of 1 m from the ground.

**Fig. 12:**
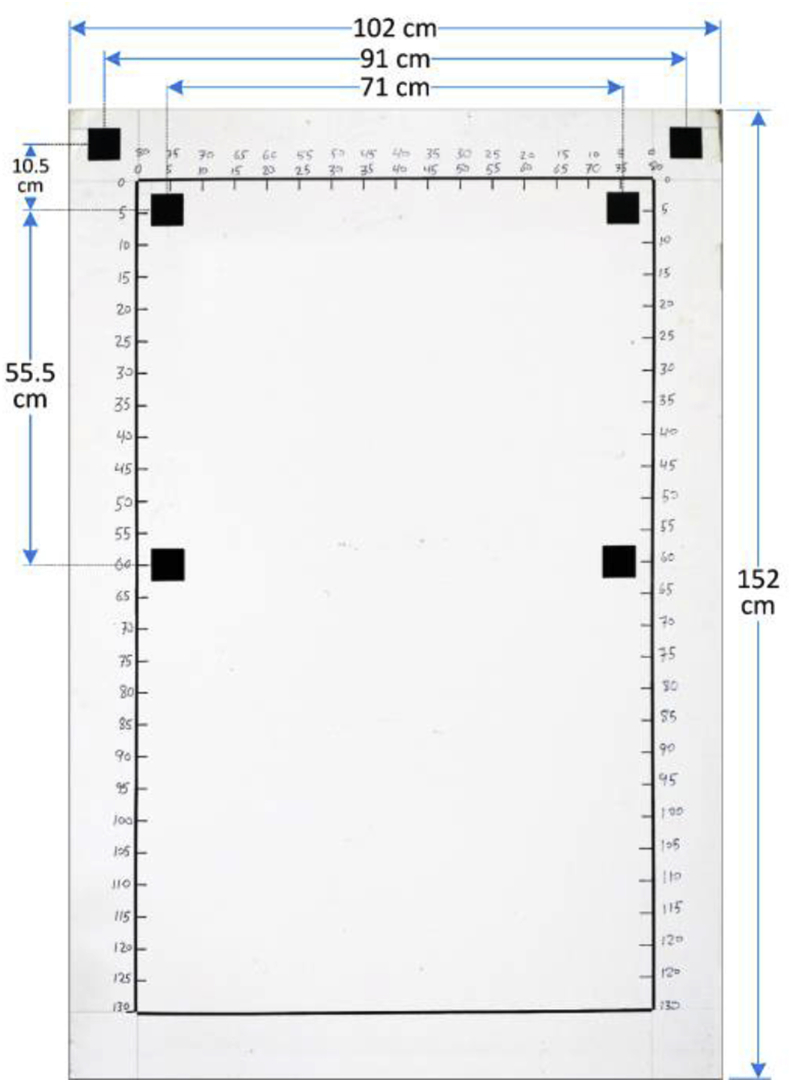
The Dimensions of the graph paper, showing the distances between the centers of the reference shapes.

**Fig. 13:**
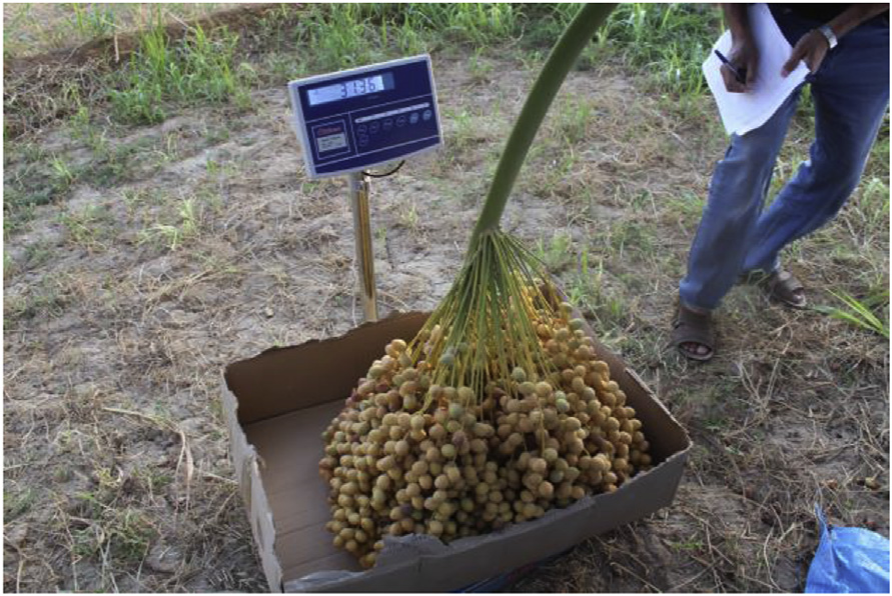
Calculate the weight of a date bunch after harvesting.

Immediately after harvesting, the date bunches in dataset-2 were placed in front of the white graph paper, as in Fig. 6, and their images were captured. Camera-1 was directed to the center of the date bunch and placed approximately 1 m away from the bunch. The graph paper was 1.52 × 1.02 m in dimensions. The dimensions of the reference objects (black squares) were 50 × 50 mm. The distances between the reference objects are illustrated in Fig. 12. Finally, the weights of these date bunches were recorded using a weight scale (Citizen, CTB 300, Metuchen, United States). The scale was placed on an even surface and date bunches were placed in a container and weighted, as shown in Fig. 13.

**Fig. 14:**
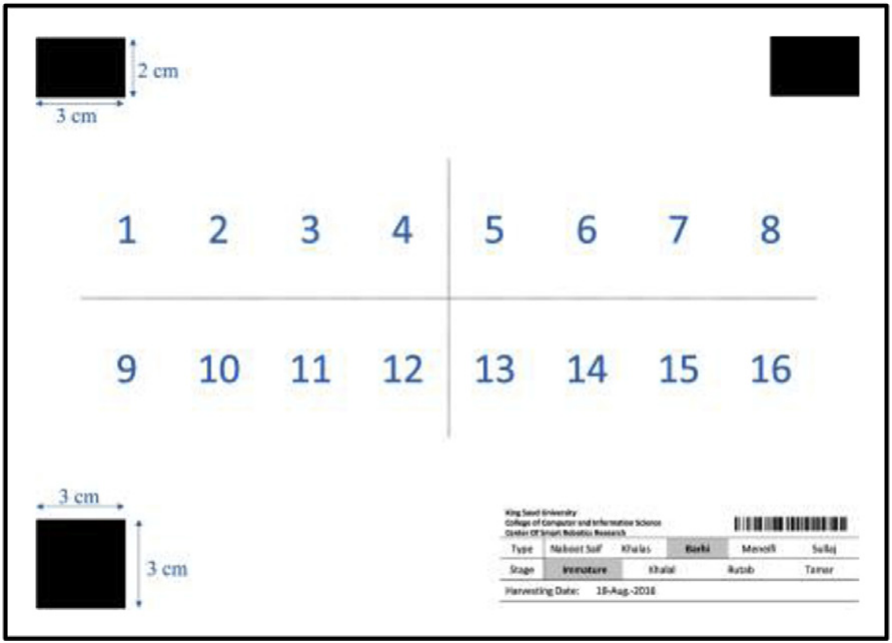
The template of reference paper used in the individual date imaging.

**Fig. 15:**
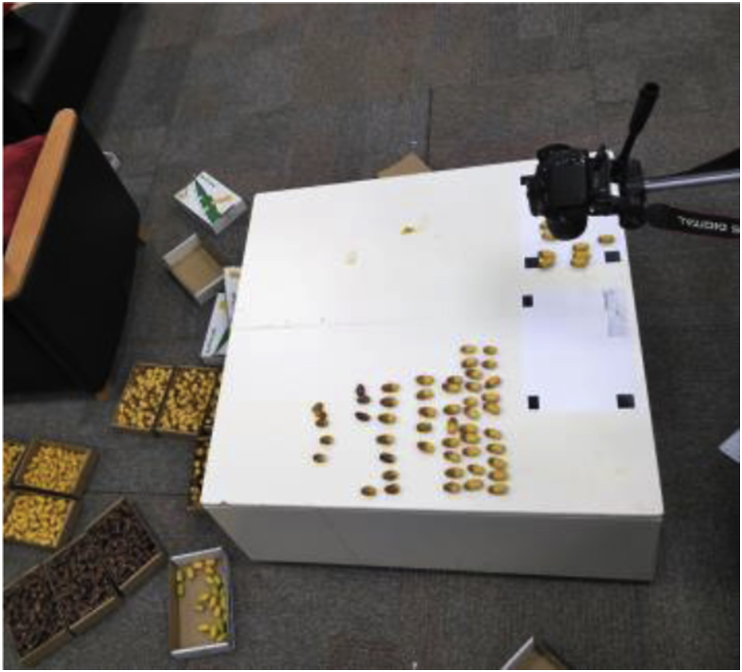
The camera position setup for the individual date imaging.

**Fig. 16:**
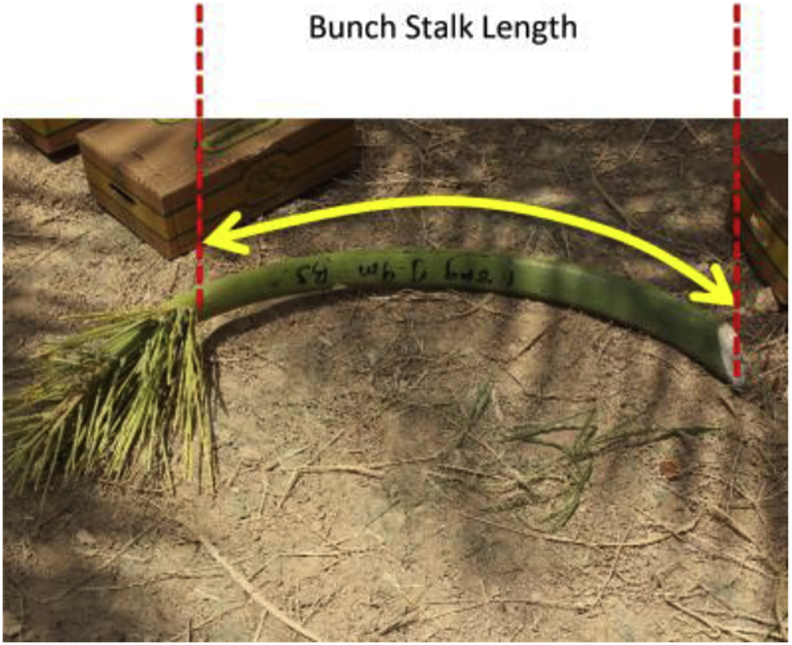
Date bunch stalk showing the ends we used to measure the length.

The images of individual dates were acquired under laboratory conditions at 18 °C. For each image, 16 dates in one maturity stage were placed above a white reference paper numbered from the top left side, as illustrated in Fig. 14. Camera-1 was directed to the center of the reference paper at a distance of 320 mm, as shown in Fig. 15. The uniform background was used to facilitate image segmentation, and the reference shapes at the paper corners can be used to estimate the dimensions and area of the dates. The dimensions of the two upper shapes were 30 × 20 mm and that of the bottom shape was 30 × 30 mm. The date information, i.e., date variety, maturity stage, harvesting date, and data code, were positioned in the bottom right of the paper. The average weights of individual dates were recorded at three maturity stages (Khalal, Rutab, and Tamar) based on around 200 dates for each stage using a Bizerba scale (model BS 200, Balingen, Germany). The average bunch stalk weight per centimeter was calculated by measuring the length and weight of ten bunch stalks. The physical length of the stalk (along the stalk curve) was measured from the inner end of the date bunch to the endpoint of the stalk, as in Fig. 16. However, the endpoint of the stalk depends on the cutting position of the bunch, and it is not the ultimate end to the stalk.

### 2.1 Dataset coding

In this dataset, we arranged the data (dates, bunches, and palms and their related visual data, i.e., image/video files, or numerical data, e.g., weight), with a coding scheme to simplify referring, linking, and facilitating future extensions of the dataset.

**Fig. 17:**
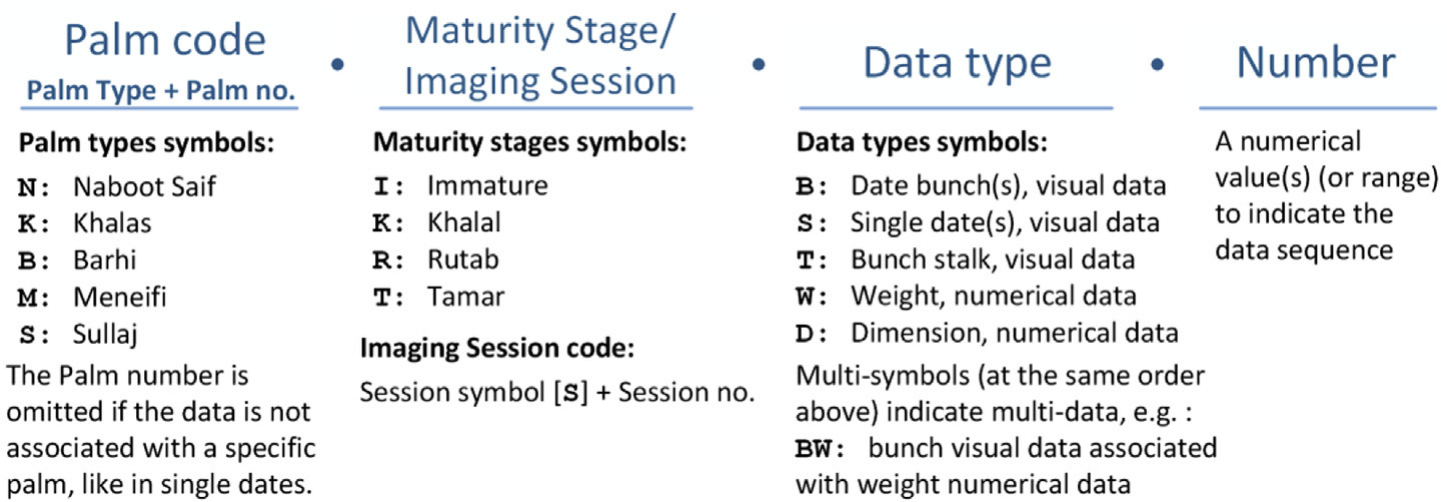
A description of the symbols used in the dataset coding.

The coding scheme consists of four parts separated by dots, as shown in Fig. 17. The first part is the code for the palm tree, which consists of two parts: palm type (variety) and palm number. The palm number is omitted if the data is not associated with a specific palm (e.g. in the case of individual dates). The second part usage depends on the dataset. In dataset-2, this part consists of a character that refers to the maturity stage of the dates (I: Immature, K: Khalal, R: Rutab, and T: Tamar). In the dataset-1, this part refers to the imaging session code, which consists of a session symbol [S] and session number, e.g., S1 refers to the first imaging session. The imaging session was used in dataset-1 instead of referring to the maturity stage explicitly because this dataset was collected over several periods of time during the maturity development of the fruit. Therefore, in one period (imaging session) the dates may have multiple maturity stages (some dates ripen before others). The third part contains one or more symbols that refer to the type of data, as stated in Fig. 17. Multiple symbols in this part refer to multi-data, e.g., BW refers to a visual bunch data that also has a weight associated with it. The combination of these symbols should be in the same order as given in Fig. 17, starting with visual data symbols (B, S, T) followed by numerical data symbols (W, D). The fourth part consists of a numerical value that indicates the data sequence. In the single date images, where the image contains 16 dates (Fig. 10), this part refers to the range sequence, e.g., 1–16.

### 2.2 General statistic of the dataset

**Fig. 18:**
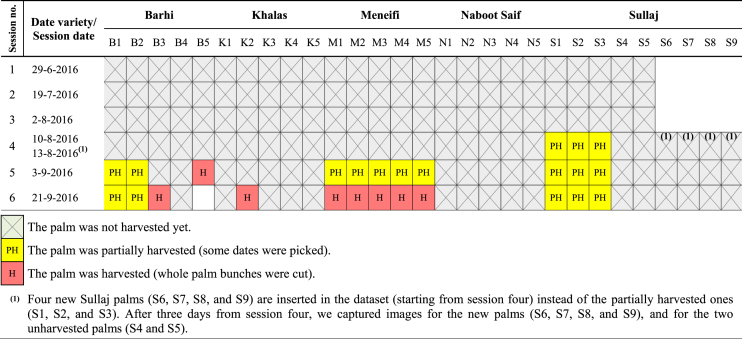
The state of each palm during the six imaging sessions: harvested, partially harvested, or not harvested.

In dataset-1, Fig. 18 gives details of the state of each palm during the six imaging sessions: harvested (whole palm bunches were cut), partially harvested (some individual Rutab dates were picked), or not harvested yet. The figure presents 29 date palms, 25 palms (5 palms per variety) were studied during all imaging sessions and four palms of Sullaj dates (S6, S7, S8, and S9) were inserted to the dataset starting from session four. This figure shows that some date palms were completely harvested before the Tamar stage (palms B3, B5, K2, M1, M2, M3, M4, and M5), and some date palms were gradually harvested over several sessions, by picking individual mature dates over several days, e.g. B1 and B2. Table 2 shows the details of the number of images taken per session for all date varieties.

**Table 2:** The number of images taken per session for all date varieties in the dataset-1.

Session no.	Session date	Barhi	Khalas	Meneifi	Naboot Saif	Sullaj	Total per session
1	29-6-2016	219	190	152	124	188	**873**
2	19-7-2016	193	161	183	245	262	**1044**
3	2-8-2016	330	256	298	156	351	**1391**
4	10-8-2016	719	425	445	401	530	**2973**
	13-8-2016	–	–	–	–	453	
5	3-9-2016	284	263	217	286	232	**1282**
6	21-9-2016	68	91	4	212	141	**516**
**Total per date variety**		**1813**	**1386**	**1299**	**1424**	**2157**	**8079**

In dataset-2, the information of the date palms and their bunches are included in an excel file. This file consists of several tables. Each table provides information about one palm tree, which includes palm variety, code, height, trunk circumference, number of bunches, weight of bunches, harvesting date, and the state of the recording. Table 3 presents overall information for all date palms in the dataset-2. Thirteen palms of the Barhi date were included in this dataset (152 bunches). Nine of them (palms 3 to 11) have full information about the palms and their bunches (all bunches in the palm). Table 4 shows sample data for one palm (B5.K.BW).

**Table 3:** List of all date palms in the dataset-2.

Palm no.	Palm code	Harvesting date	Total palm yield (kg)	Palm height (m)	Palm trunk circumference (m)	Total no. of bunches in the palm		Number of bunches			
							weighted	Recorded still image		Marked on palm	Recorded video
								On graph paper	On Palm		
1	B1.K.BW	16/8/2016	–	–	–	12	3	3	3	0	0
2	B2.K.BW	16/8/2016	373.5	–	–	15	15	15	14	0	0
3	B3.K.BW	18/8/2016	396.1	2.85	1.95	15	15	15	15	15	15
4	B4.K.BW	19/8/2016	222.2	2.6	1.77	12	12	12	12	12	12
5	B5.K.BW	21/8/2016	287.5	2.75	1.74	14	14	14	14	14	14
6	B6.K.BW	26/8/2016	361.0	3.7	1.98	15	15	15	15	15	15
7	B7.K.BW	20/8/2016	312	3.3	1.78	13	13	13	13	13	13
8	B8.K.BW	19/8/2016	307.8	3.45	1.72	14	14	14	14	14	14
9	B9.K.BW	20/8/2016	375.9	4.85	2.15	14	14	14	14	14	14
10	B10.K.BW	26/8/2016	203.0	1.72	1.85	11	11	11	11	11	11
11	B11.K.BW	21/8/2016	227.1	1.72	1.84	12	12	12	12	12	12
12	B12.K.BW	17/8/2016	–	–	–	–	4	4	2	0	0
13	B13.K.BW	18/8/2016	100.8	–	–	10	10	10	6	0	0
**Total (Number of bunches)**							152	152	145	120	120

**Table 4:** An example of data for a date palm in the dataset-2.

Palm variety:	Barhi
Palm code	B5.K.BW
Palm height	2.75 m
Palm trunk circumference	1.74 m
No. of bunches in the palm	14
Harvesting date	21/8/2016

No.	Bunch code	Bunch weight (kg)	Recording state of the bunch			
			Image on palm	Image on graph paper	Video	Marked on palm
1	B5.K.BW.46	9.68	yes	yes	yes	yes
2	B5.K.BW.47	17.66	yes	yes	yes	yes
3	B5.K.BW.48	11.36	yes	yes	yes	yes
4	B5.K.BW.49	13.98	yes	yes	yes	yes
5	B5.K.BW.50	27.52	yes	yes	yes	yes
6	B5.K.BW.51	20.52	yes	yes	yes	yes
7	B5.K.BW.52	29.90	yes	yes	yes	yes
8	B5.K.BW.53	27.50	yes	yes	yes	yes
9	B5.K.BW.54	13.44	yes	yes	yes	yes
10	B5.K.BW.55	26.22	yes	yes	yes	yes
11	B5.K.BW.56	30.86	yes	yes	yes	yes
12	B5.K.BW.57	14.08	yes	yes	yes	yes
13	B5.K.BW.58	28.60	yes	yes	yes	yes
14	B5.K.BW.59	16.16	yes	yes	yes	yes

In the current dataset, images of 256 individual dates of Sullaj, Barhi, and Meneifi dates were captured, as shown in Table 5. The average weights of individual dates were recorded for Sullaj and Barhi dates based on 1740 dates, Table 6 presents samples of Barhi dates. The length and weight of the ten bunch stalks for Barhi dates were measured as presented in Table 7.

**Table 5:** The number of individual dates in dataset-2.

		Maturity stage				Total
		Immature	Khalal	Rutab	Tamar	
No. of dates	Sullaj	16	32	80^a^	32	160
	Barhi	16	16	16	16	64
	Meneifi	–	–	16	16	32

a The horizontal and vertical diameters were measured manually for 16 dates of this variety (S.R1.SD.17–32) using caliper.

**Table 6:** Samples of single date weights for the Barhi variety in three maturity stages: Khalal, Rutab, and Tamar.

Maturity Stage	Khalal	Rutab	Tamar
Harvesting date	18-Aug.-2016	3-Sep.-2016	18-Aug.-2016
No. of dates	180	160	200
Weight (kg)	1.855	1.885	1.345
image

**Table 7:** The length and weight measurements of Barhi date bunch stalks.

Bunch stalk code: B.K.TWD.	1	2	3	4	5	6	7	8	9	10
Weight (kg)	2.90	2.42	1.80	1.56	1.84	1.40	3.44	2.78	2.96	3.64
Length (cm)	125	103	90	94	94	82	130	136	114	167
Weight per length (g/cm)	23.2	23.5	20	16.6	19.58	17.07	26.46	20.44	25.97	21.8
